# Evolving knowledge in surgical oncology of pancreatic cancer: from theory to clinical practice—a fifteen-year journey at a tertiary referral centre

**DOI:** 10.1007/s13304-022-01346-x

**Published:** 2022-08-25

**Authors:** Riccardo Casadei, Claudio Ricci, Carlo Ingaldi, Laura Alberici, Emilio De Raffele, Bianca Barcia, Cristina Mosconi, Margherita Diegoli, Mariacristina Di Marco, Giovanni Brandi, Rocco Maurizio Zagari, Nico Pagano, Leonardo Henry Eusebi, Carla Serra, Marina Migliori, Alessandra Guido, Donatella Santini, Francesca Rosini, Deborah Malvi, Francesco Minni

**Affiliations:** 1 Division of Pancreatic Surgery, IRCCS, Azienda Ospedaliero Universitaria di Bologna, S.Orsola-Malpighi Hospital, Via Massarenti n.9, 40138 Bologna, Italy; 2 Department of Internal Medicine and Surgery (DIMEC), Alma Mater Studiorum, University of Bologna, S.Orsola-Malpighi Hospital, Bologna, Italy; 3 Department of Radiology, IRCCS Azienda Ospedaliero-Universitaria di Bologna, Bologna, Italy; 4 Division of Gastroenterology, IRCCS Azienda Ospedaliero-Universitaria di Bologna, Bologna, Italy; 5 Medical Oncology, IRCCS Azienda Ospedaliero-Universitaria di Bologna, Bologna, Italy; 6 Division of Pathologhy, Azienda Ospedaliero-Universitaria di Bologna, Bologna, Italy

**Keywords:** Pancreatic ductal adenocarcinoma, Incidence, Diagnosis, Treatment, Survival

## Abstract

Pancreatic ductal adenocarcinoma (PDAC) is an increasing disease having a poor prognosis. The aim of the present study was to evaluate the effect of different models of care for pancreatic cancer in a tertiary referral centre in the period 2006–2020. Retrospective study of patients with PDAC observed from January 2006 to December 2020. The demographic and clinical data, and data regarding the imaging techniques used, preoperative staging, management, survival and multidisciplinary tumour board (MDTB) evaluation were collected and compared in three different periods characterised by different organisation of pancreatic cancer services: period A (2006–2010); period B (2011–2015) and period C (2016–2020). One thousand four hundred seven patients were analysed: 441(31.3%) in period A; 413 (29.4%) in B and 553 (39.3%) in C. The proportion of patients increased significantly, from 31.3% to 39.3% (*P* = 0.032). Body mass index (*P* = 0.033), comorbidity rate (*P* = 0.002) and Karnofsky performance status (*P* < 0.001) showed significant differences. Computed tomography scans (*P* < 0.001), endoscopic ultrasound (*P* < 0.001), fine needle aspiration, fine needle biopsy (*P* < 0.001), and fluorodeoxyglucose-positron emission tomography/computed tomography (*P* < 0.001) increased; contrast-enhanced ultrasound (*P* = 0.028) decreased. The cTNM was significantly different (*P* < 0.001). The MDTB evaluation increased significantly (*P* < 0.001). Up-front surgery and exploratory laparotomy decreased (*P* < 0.001), neoadjuvant treatment increased (*P* < 0.001). The present study showed the evolving knowledge in surgical oncology of pancreatic cancer at a tertiary referral centre over the time. The different models of care of pancreatic cancer, in particular the introduction of the MDTB and the institution of a pancreas unit to the decision-making process seemed to be influential.

## Introduction

Approximately 60,430 and 14,263 new diagnoses of pancreatic cancer were estimated in the U.S. and Italy in 2021, respectively, with an increasing incidence rate of 0.5–1% per year [1, 2]. Based on GLOBOCAN 2018 estimates, pancreatic cancer accounted for 458,918 new cases and caused 432,242 deaths (4.5% of all deaths caused by cancer) in 2018 [3]. The 5-year survival rate was approximately 10% for the first time in 2020, and pancreatic cancer is projected to become the second-leading cause of cancer death in the U.S. by 2030 [4, 5]. Much effort is being made to resolve this alarming situation. Imaging resolution has been improved by enhanced computed tomography with a high-quality pancreatic protocol and by the introduction of endoscopic ultrasound [6]. The accurate assessment of surgical indications, techniques, and perioperative care have been very useful regarding better short-term results. Furthermore, adjuvant and neoadjuvant radiochemotherapy, and immunotherapies were more frequently incorporated into multimodal treatment [7, 8] to obtain better long-term results and, finally, consensus recommendations for improving pancreatic cancer care were adopted [9]. Nationwide trends regarding the incidence, treatment and survival of pancreatic cancer patients to adequately measure the impact of these improvements in the management of pancreatic cancer have been reported in only a few studies [10–18]. To the Authors’ knowledge, there are no studies which evaluate the changes in trends regarding pancreatic ductal adenocarcinoma in a tertiary referral centre over the time periods. The aim of the present study was to evaluate the effect of different models of care for pancreatic cancer by analysing the changes in trends of the demographic and clinical data, the imaging techniques used, the management adopted and long-term survival in a tertiary referral centre in the period 2006–2020.

## Methods

### Study design

This was a retrospective study of patients affected by PDAC observed at S. Orsola-Malpighi Hospital, Bologna, Italy, from January 2006 to December 2020. The study was approved by the Ethical Committee of S. Orsola-Malpighi Hospital (code: 642017 U/Oss), and patient informed consent was obtained from all the participants enrolled in the study. Patients were included in this study if they provided a consent to include their anonymized data in future research. All patients with an ICD-9-CM code diagnosis of 157.0, 157.1, 157.2, 157.8, and 157.9, and ICD-9-CM procedures 52.51, 52.52, 52.53, 52.6, and 52.7 were extracted and entered into the database for pancreatic diseases (PANBO, code: 064/2017/U/OSS). Patients were included in this study only when admitted to S. Orsola-Malpighi hospital. Patients seen only in outpatient clinics were not included. The following data were collected for each patient: (1) demographic and clinical; gender, age, co-morbidities, body mass index (BMI), symptoms (back pain, diabetes, weight loss, jaundice, stenting), Ca 19–9 serum value and Karnofsky performance status (KPS); (2) imaging techniques used: thoraco-abdominal computed tomography (CT) scan, magnetic resonance (MR), endoscopic ultrasound (EUS), contrast-enhanced ultrasound (CEUS), fluorodeoxyglucose-positron emission tomography/computed tomography (FDG-PET/CT), and fine needle aspiration (FNA) or fine needle biopsy (FNB), and 3) preoperative staging, management and survival; site of the tumour (head/uncinate process, body/tail and multiple), cTNM according to the American Joint Committee on Cancer AJCC 8° edition [19], National Comprehensive Cancer Network (NCCN) classification [20] (resectable, borderline resectable, locally advanced and metastatic), multidisciplinary tumour board (MDTB) evaluation, type of management (up-front surgery, palliative care, neoadjuvant approach), surgical resection, unnecessary surgery and overall survival. Using these data, three different periods were identified and compared: period A (2006–2010); period B (2011–2015) and period C (2016–2020). The three different periods were characterised by the different organisation of pancreatic cancer services. In the period 2006–2010, the diagnostic and therapeutic decision-making process was related only to common evidence-based recommendations regarding pancreatic cancer. The decisions were usually made by a single medical doctor or a single team of specialists (for example, surgeons). However, in this period there was a multidisciplinary tumour board but it played a modest role in the decision-making process because it was not recognized by our Hospital. In the period 2011–2015 integrated healthcare policies promoted specialisation and put a multidisciplinary tumour board (MDTB) at the centre of the decision-making process. The MDTB consisted of several specialists with expertise in the field of pancreatic cancer (surgeons, gastroenterologists, radiologists, oncologists and radiation oncologists, pathologists, endoscopists and diabetologists). In this period, the diagnostic and therapeutic decision-making process was entrusted to the MDTB. All decisions were made by the MDTB, and a report was issued. Finally, in the third period, from 2016 to 2020, healthcare policies included the MDTB and a Pancreas Unit. The Authors’ Pancreas Unit was equipped with a dedicated surgical team and a hospital ward, and was able to guarantee the different specialties necessary for performing pancreatic surgery: gastroenterology, interventional radiology, digestive endoscopy, diagnostic radiology, nuclear medicine, diabetology, intensive care unit (ICU), pain medication, nutritional medicine, psychosocial oncology and pathology units with proven experience in pancreatic diseases.

### Statistical analysis

All the categorical variables were described as frequencies and percentages while the continuous variables were reported as median or means, interquartile range (IQR) or standard deviation (SD). Comparison of the three groups was carried out using the Fischer’s exact test, Student’s *t* test and Pearson chi square test. Two-tailed *P* values less than 0.05 were considered statistically significant. All statistical analyses were carried out by running the Statistical Package for the Social Sciences (SPSS, Chicago, IL), version 13 on a personal computer.

## Results

**Fig. 1:**
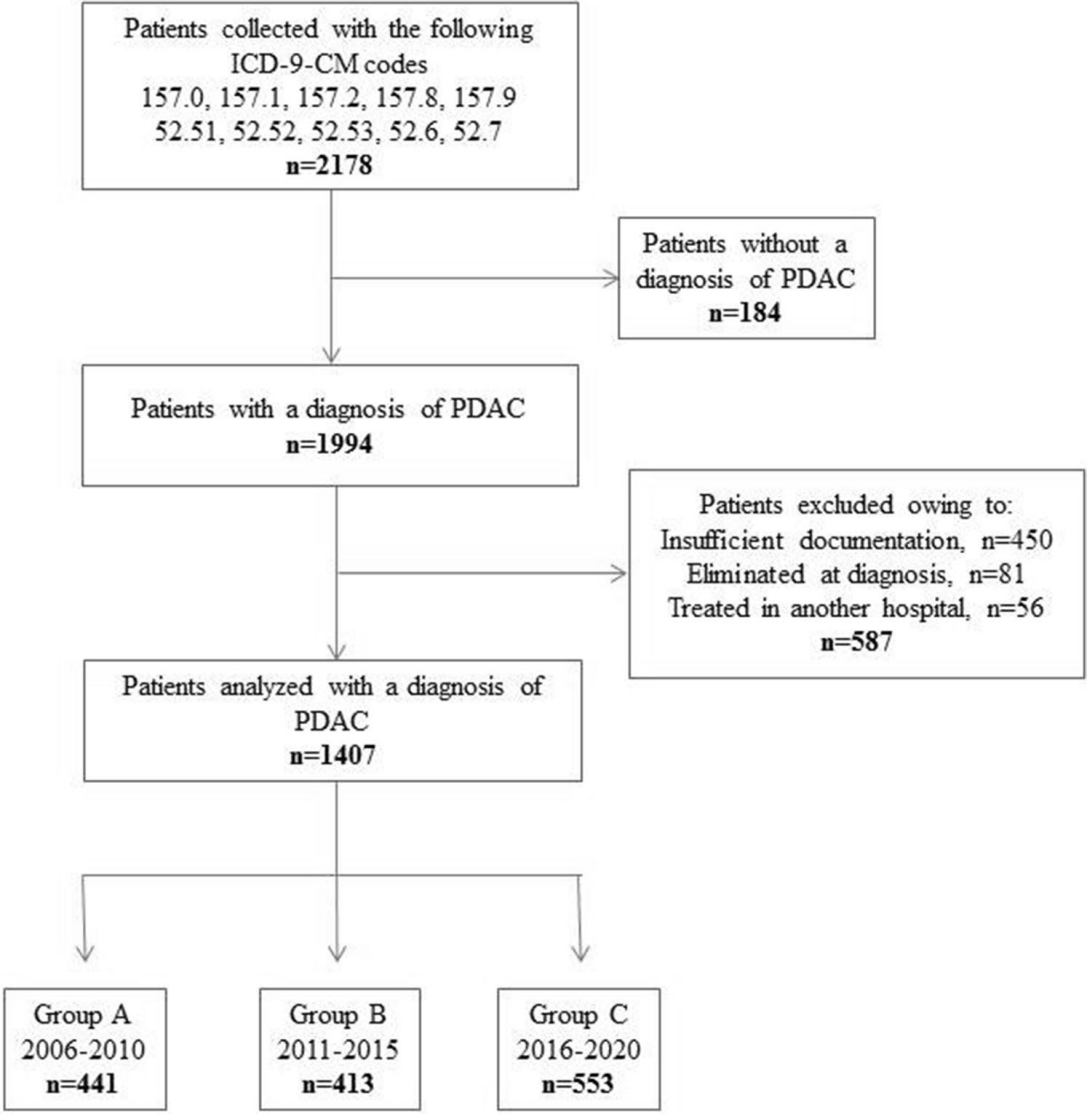
Flowchart of the selection process of the patients enrolled by pancreatic ductal adenocarcinoma (PDAC) observed from 2006 to 2020. *PDAC* pancreatic ductal adenocarcinoma

From January 2006 to December 2020, a total of 2178 patients with a suspected diagnosis of pancreatic cancer were enrolled. Of the latter, 771 were excluded: 184 were without a diagnosis of PDAC, 450 had insufficient documentation (lack of key imaging studies, in particular for patients from other countries), 81 were eliminated at diagnosis (patients who did not receive any treatment or who were lost to follow-up soon after diagnosis) and 56 had been treated in another hospital. In total, the patients having a diagnosis of PDAC were 1407: 441(31.3%) in period A (2006–2010). 413 (29.4%) in period B (2011–2015), and 553 (39.3%) in C (2016–2020) (Fig. 1).

The demographic and clinical characteristics of the patients affected by PDAC in the three periods analysed are summarised in Table 1. The proportion of patiens affected by PDAC admitted to our Hospital increased significantly over the time periods, from 31.3% (2006–2010) to 39.3% (2016–2020) (*P* = 0.032). There were no statistically significant differences regarding gender, age, symptoms, stenting and Ca 19–9 serum value. On the contrary, the BMI (period A = 23.9 kg/m^2^; period B = 24.3 kg/m^2^ and period C = 24.4 kg/m^2^; *P* = 0.033), comorbidity rate (period A = 75.5%; period B = 85.0% and C = 80.3%; *P* = 0.002) and KPS (period A = 78.2%, period B = 74.5%, and period C = 79.2%; *P* < 0.001). showed significant differences in the three periods.

**Table 1:** Demographic and clinical characteristics of the patients affected by pancreatic ductal adenocarcinoma (*n* = 1407) in the three periods analysed

Parameters	Total *N* (%)	Group A	Group B	Group C	*P* value
	Mean (SD)	2006–2010	2011–2015	2016–2020	
Gender					0.274
Male	738 (52.5)	244 (55.3)	206 (49.9)	288 (52.1)	
Female	669 (47.5)	197 (44.7)	207 (50.1)	265 (47.9)	
Age, (years)	72.1 (11.5)	70.7 (10.9)	71.3 (11.7)	72.1 (11.7)	0.082
BMI (Kg/m^2^)*	24.2 (4.2)	23.9 (4.1)	24.3 (4.2)	24.4 (4.5)	**0.033**
Comorbidity					**0.002**
No	279 (19.8)	108 (24.5)	62 (15)	109 (19.7)	
Yes	1128 (80.2)	333 (75.5)	351 (85)	444 (80.3)	
Symptoms					0.463
No	509 (36.2)	158 (35.8)	141 (34.1)	210 (37.9)	
Yes	898 (63.8)	283 (64.2)	272 (65.9)	343 (62.1)	
Back pain					0.084
No	1172 (83.3)	368 (83.5)	331 (80.2)	473 (85.5)	
Yes	235 (16.7)	73 (16.5)	82 (19.8)	80 (14.5)	
Diabetes					0.114
No	1071 (76.2)	323 (73.2)	312 (75.5)	436 (78.8)	
Yes	336 (23.8)	118 (26.8)	101 (24.5)	117 (21.2)	0.906
Weight loss					
No	968 (68.8)	304 (68.9)	287 (69.5)	377 (68.2)	
Yes	439 (31.2)	137 (31.1)	126 (30.5)	176 (31.8)	
Jaundice					0.077
No	777 (55.2)	232 (52.6)	219 (53)	326 (58.9)	
Yes	630 (44.8)	209 (47.4)	194 (47)	227 (41.1)	
Stent					0.178
No	864 (61.4)	258 (58.5)	251 (60.7)	355 (64.2)	
Yes	543 (38.6)	183 (41.5)	162 (39.3)	198 (35.8)	
Ca 19.9 (U/mL)^	500 (90–3142)	493 (91–3110)	559 (83–3412)	468 (92–2960)	0.931
KPS	77.5 (16.6)	78.2 (16.7)	74.5 (17.9)	79.2 (14.9)	< 0.001
Total	1407	441 (31.3)	413 (29.4)	553 (39.3)	0.032

The bold values represent the significant values

*SD* Standard Deviation, *BMI* Body Mass Index, *KPS* Karnofsky Performance Status

**Fig. 2:**
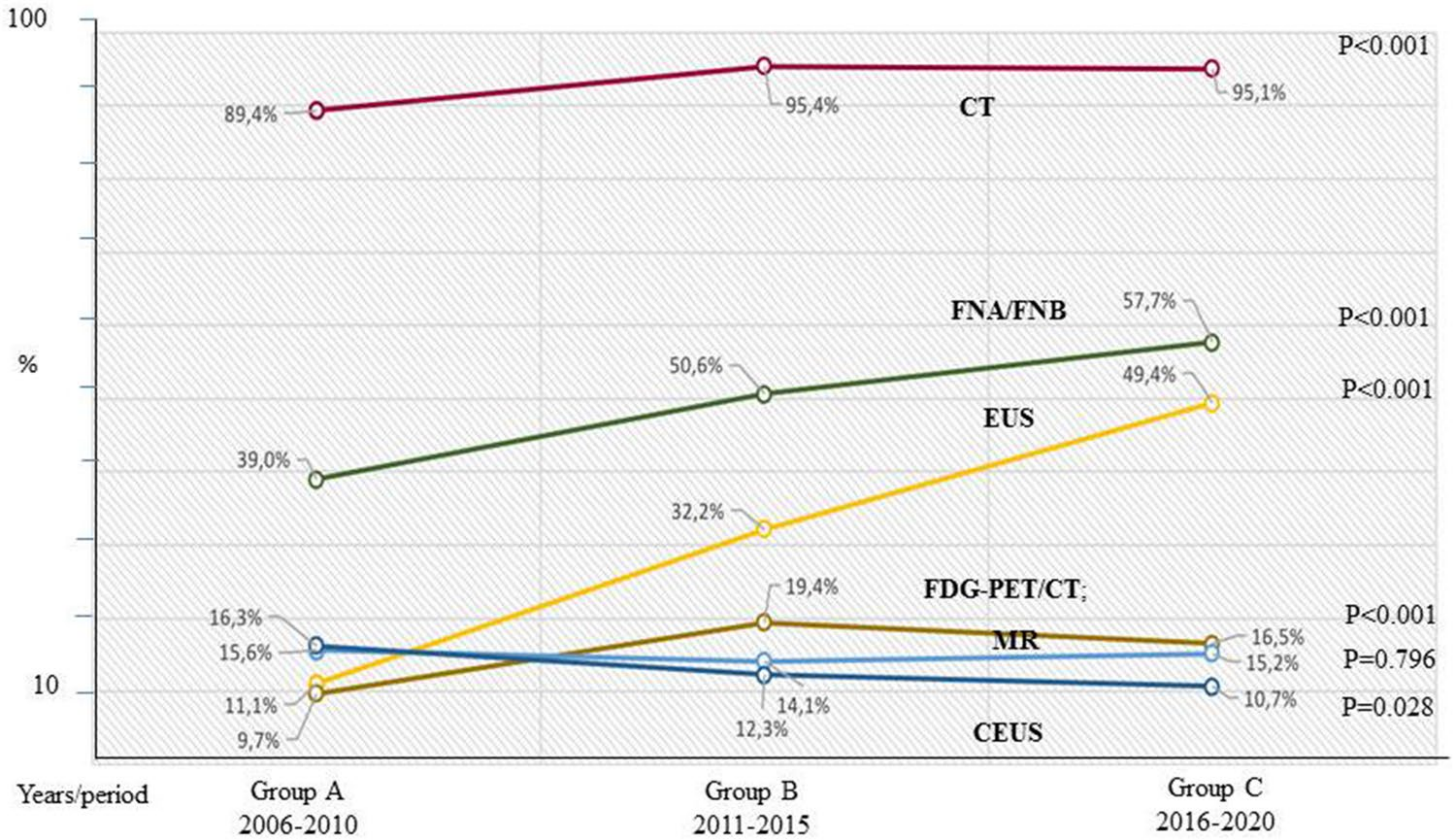
Changes in trends of the imaging techniques in the three periods analysed. *CT* Computed Tomography, *FNA/FNB* Fine Needle Aspiration/Biopsy, *EUC* Endoscopic ultrasound, *FDG-PET/CT* Fluoro DeoxyGlucose-Positron Emission Tomography/Computed Tomography, *MR* Magnetic Resonance, *CEUS* Contrast Enhanced Ultrasound

Table 2 and Fig. 2 described the percentages of the imaging techniques used in the three periods analysed. A CT scan (from 89.4% in period A to 95.4% in period B to 95.1% in period C (*P* < 0.001), EUS (from 11.1% in period A to 32.2% in period B to 49.4% in period C (*P* < 0.001), FNA/FNB (from 39.0% in period A to 50.6% in period B to 57.7% in period C (*P* < 0.001), FDG-PET/CT (from 9.7% in period A to 19.4% in period B to 16.5% in period C (*P* < 0.001) showed a significantly increasing rate over the time periods. On the contrary, CEUS (from 16.3% in period A to 12.3% in period B to 10.7% in period C (*P* = 0.028) showed a significantly decreasing rate over the time periods.

**Table 2:** Trends of the imaging techniques in the three periods analysed

Imaging techniques	Total *N* (%)	Group A	Group B	Group C	*P* value
	Median (IQR)	2006–2010	2011–2015	2016–2020	
CT scan					< 0.001
No	93 (6.6)	47 (10.6)	19 (4.6)	27 (4.9)	
Yes	1314 (93.4)	394 (89.4)	394 (95.4)	526 (95.1)	
MR					0.796
No	1196 (85.0)	372 (84.4)	335 (85.9)	469 (84.8)	
Yes	211 (15.0)	69 (15.6)	58 (14.1)	84 (15.2)	
EUS					** < 0.001**
No	952 (67.6)	392 (88.9)	280 (67.8)	280 (50.6)	
Yes	455 (32.4)	49 (11.1)	133 (32.2)	273 (49.4)	
CEUS					**0.028**
No	1125 (87.1)	369 (83.7)	362 (87.7)	494 (89.3)	
Yes	182 (12.9)	72 (16.3)	51 (12.3)	59 (10.7)	
FDG-PET/CT					** < 0.001**
No	1193 (84.8)	398 (90.3)	333 (80.6)	462 (83.5)	
Yes	214 (15.2)	43 (9.7)	80 (19.4)	91 (16.5)	
FNA/FNB					** < 0.001**
No	707 (50.3)	269 (61)	204 (49.4)	234 (42.3)	
Yes	700 (49.7)	172 (39)	209 (50.6)	319 (57.7)	
Total	1407	441 (31.3)	413 (29.4)	553 (39.3)	

The bold values represent the significant values

*IQR* interquartile range, *CT* Computed Tomography, *MR* Magnetic Resonance, *EUS* Endoscopic UltraSound, *CEUS* Contrast Enhanced UltraSound, *FDG-PET/CT* FluoroDeoxyglucose-Positron Emission Tomography/Computed Tomography, *FNA/FNB* Fine Needle Aspiration/Biopsy

**Fig. 3:**
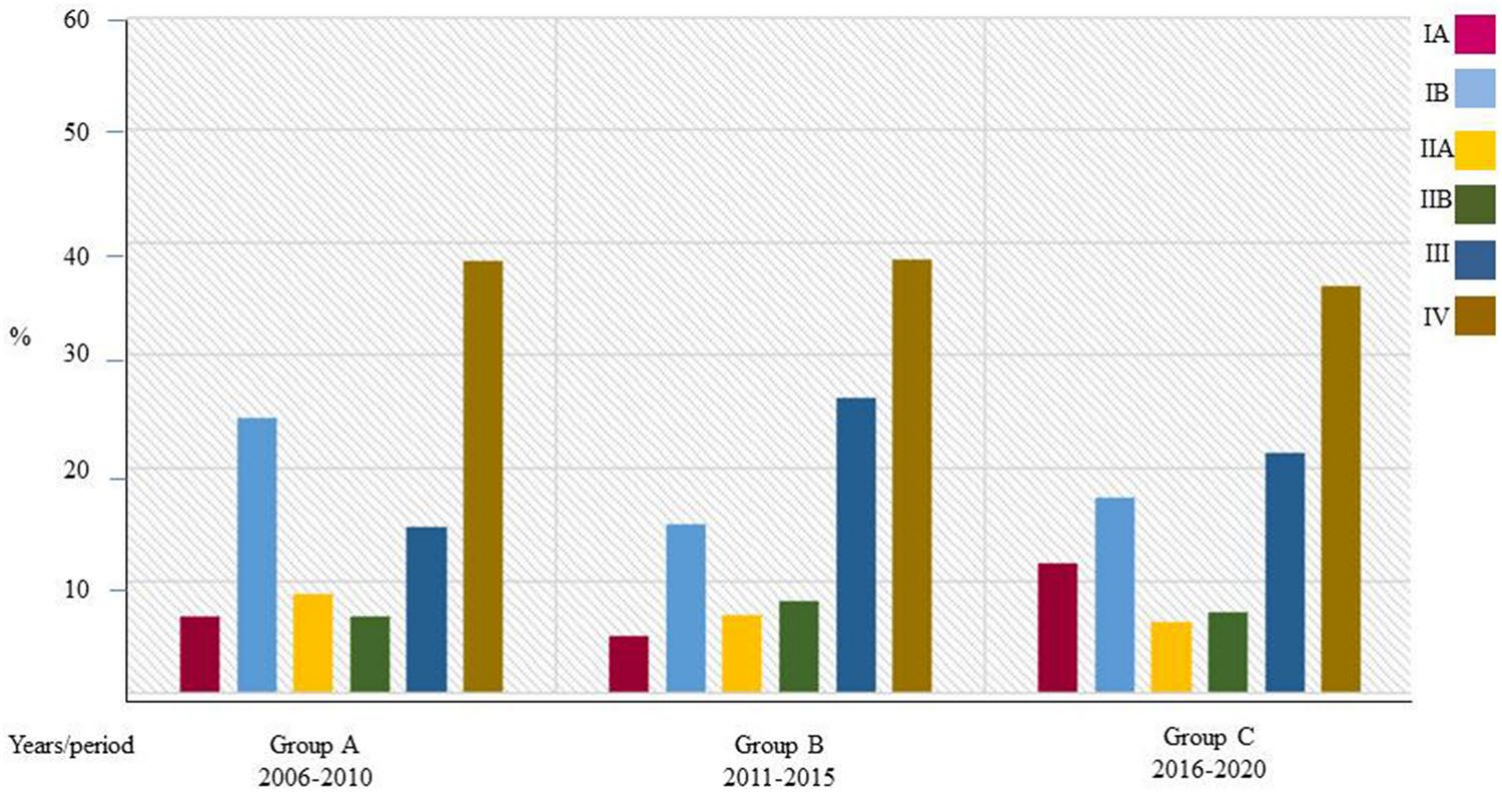
Incidence of cTNM staging of patients affected by pancreatic ductal adenocarcinoma in the three period analysed

Table 3 reports the differences regarding preoperative staging, type of management and overall survival. Figure 3 shows only the differences regarding preoperative staging. The cTNM was significantly different in the three periods (*P* < 0.001). In particular, stage IA increased from period A (6.8%) to period C (11.6%).

**Table 3:** The staging, management and survival of patients affected by pancreatic ductal adenocarcinoma (*n* = 1407) in the three periods analysed

Parameters	Total *N* (%)	Group A	Group B	Group C	*P* value
	Median (IQR)	2006–2010	2011–2015	2016–2020	
Site					0.251
Head/Uncinate	852 (60.6)	279 (63.3)	238 (57.6)	335 (60.5)	
Body/Tail	457 (32.5)	134 (30.4)	138 (33.4)	185 (33.5)	
Multiple	98 (6.9)	28 (6.3)	37 (9)	33 (6)	
cTNM^*^					** < 0.001**
IA	115 (8.2)	30 (6.8)	21 (5.1)	64 (11.6)	
IB	266 (18.9)	108 (24.5)	62 (15)	96 (17.4)	
IIA	103 (7.3)	39 (8.9)	29 (7)	35 (6.3)	
IIB	104 (7.4)	30 (6.8)	34 (8.2)	40 (7.2)	
III	291 (20.7)	65 (14.7)	108 (26.2)	118 (21.3)	
IV	528 (37.5)	169 (38.3)	159 (38.5)	200 (36.2)	
NCCN classification					
Resectable	520 (36.9)	161 (36.5)	149 (36.1)	210 (37.9)	
Borderline	202 (14.4)	74 (16.8)	51 (12.4)	77 (13.9)	
Locally advanced	157 (11.2)	37 (8.4)	54 (13)	66 (11.9)	
Metastatic	528 (37.5)	169 (38.3)	159 (38.5)	200 (36.3)	
MDTB evaluation					** < 0.001**
No	787 (57.5)	297 (67.4)	273 (66.1)	226 (41.7)	
Yes	581 (42.5)	144(32.6)	134 (33.9)	316 (58.3)	
Initial therapeutic decision making					** < 0.001**
Up-front surgery	442 (31.4)	173 (39.2)	122 (29.5)	147 (26.6)	
Neoadjuvant	107 (7.6)	28 (6.4)	24 (5.8)	55 (9.9)	
Palliative care	858 (61.0)	240 (54.4)	267 (64.7)	351 (63.5)	
Surgical resection					0.339
No Yes	1047 (74.4) 360 (25.6)	317 (71.9) 124 (28.2)	312 (75.5) 101 (24.5)	418 (75.6) 135 (24.4)	
R status					
R0 R1 R2	193(53.6) 162(45.0) 5(1.4)	82(66.1) 41(33.1) 1(0.8)	65(64.4) 34(33.7) 2(1.9)	46(34.1) 87(64.4) 2(1.5)	** < 0.001**
Mini-invasive approach					
No Yes	337(93.6) 23(6.4)	124(100) 0(0.0)	94(93.1) 7(6.9)	119(88.1) 16(11.9)	** < 0.001**
Exploratory laparotomy					** < 0.001**
No	1296 (92.1)	388 (88)	381 (92.3)	527 (95.3)	
Yes	111 (7.9)	53 (12)	32 (7.7)	26 (4.7)	
Overall Survival (months)	8.3 (3–19)	7 (3–17)	7 (3–17)	10 (4–20)	0.097
Total	**1407**	**441 (31.3)**	**413 (29.4)**	**553 (39.3)**	

The bold values represent the significant values

*IQR* interquartile range**, ***NCCN* National Comprehensive Cancer Network, *MDTB* MultiDisciplinary Tumour Board

*cTNM according to the AJCC 8th edition [19]

The MDTB evaluation increased significantly from period A (32.6%) to period C (58.3%) (*P* < 0.001). First, therapeutic decision making changed significantly over the time periods (*P* < 0.001). In particular, up-front surgery decreased from 39.2% in period A to 26.6% in period C while the neoadjuvant approach increased from 6.4% in period A to 9.9% in period C. The resection rate of the entire sample was 25.6% with only slight differences among the three periods (28.2% in period A, 24.5% in period B and 24.4% in period C; *P* = 0.339). R status changed significantly over the time (*P* < 0.001). In particular in the last period, the R0 status decreased significantly respect to the two previous period (34.1% versus 66.1 and 64.1%, respectively). Mini-invasive laparoscopic approach increased significantly over the time from 0% of the period 2006–2010 to 11.9% of the period 2016–2020 (*P* < 0.001). Exploratory laparotomy decreased significantly from period A (12.0%) to the period C (4.7%) (*P* < 0.001). Median overall survival was 8.3 months (3–19) and it did not significantly change over the time periods (period A = 7 months, period B = 7 months and period C = 10 months; *P* = 0.097). Table 4 reported the postoperative outcomes after pancreatic resection for PDAC. Overall morbidity, 30-days mortality, 90-days mortality, length of stay (LOS) were not significantly different in the three periods. CR-POPF resulted significantly different over the time. In particular, CR-POPF increased from period A to period C (*P* < 0.001) (grade B, from 10.6 to 22.7%; grade C, from 0 to 3.0%, respectively).

**Table 4:** Postoperative outcomes after pancreatic resection for pancreatic ductal adenocarcinoma over the time

Postoperative data	N (%) or Median (IQR)	Group A 2006–2010	Group B 2011–2015	Group C 2016–2020	*P* value
Morbidity according to CDC classification					
No Grade I Grade II Grade IIIa–IIIb Grade Iva–IVb Grade V	153 (42.5) 60 (16.7) 73 (20.3) 41 (11.4) 22 (6.1) 11 (3.1)	53 (42.7) 21 (16.9) 24 (19.4) 15 (12.1) 10 (8.1) 1 (0.8)	40 (39.6) 18 (17.8) 19 (18.8) 12 (11.9) 7 (6.9) 5 (5)	60 (44.4) 21 (15.6) 30 (22.2) 14 (10.4) 5 (3.7) 5 (3.7)	0.753
CR-POPF					
Not a risk * No/BL B C	53 (14.8) 243 (67.5) 57 (15.9) 7 (2)	0 (0.0) 111 (89.4) 13 (10.6) 0 (0.0)	24 (23.5) 67 (65.9) 9 (9.4) 1 (1.2)	23 (16.7) 78 (57.6) 30 (22.7) 4 (3.0)	** < 0.001**
LOS (in day)	12 (10–19)	13 (10–18)	12 (10–19)	12 (9–20)	0.968
30-days mortality					
No Yes	350 (97.2) 11 (3.1)	123 (99.2) 1 (0.8)	96 (95.1) 5 (4.9)	130 (96.3) 5 (3.7)	0.171
90-days mortality					
No Yes	345 (95.8) 15 (4.2)	121 (97.6) 3 (2.4)	95 (94) 6 (5.9)	129 (96.6) 6 (4.4)	0.413
Total	360	124(34.4)	101(28.1)	135(37.5)	

The bold value represents the significant value

*IQR* InterQuartile Range, *CDC* Clavien Dindo Classification, *CR-POPF* Clinically Relevant PostOperative Pancreatic Fistula, * total pancreatectomy, *BL* Biochemical Leak, *LOS* Length of Stay

## Discussion

In the present study, a total of 1407 patients with PDAC were analysed, and were compared in relation to the three periods characterised by different models of care for pancreatic cancer in a tertiary referral centre during the period 2006–2020. In the literature, there are only a few nationwide studies [10–18] which have analysed the changes in trends regarding PDAC over time. On the contrary, to the Authors’ knowledge, the present study represents the first study which analysed the changes in trends regarding PDAC together with the associated models of care for pancreatic cancer in a tertiary referral centre over a fifteen-year time period. However, there is a recent study reporting on 3000 pancreaticoduodenectomy performed at a single centre over 20 years that provided time-related changes in management of pancreatic cancer [21].

Many differences were found among the three periods in which different models of care were adopted. The epidemiological and clinical characteristics of the population, the incidence rate, the BMI, the comorbidity rate and KFS were significantly different in each of the time periods. The increasing incidence of PDAC has been reported by only a few nationwide studies [10–18]. The data from the Danish Cancer Registry [13], covering a sixty-one year period from 1943 to 2003, reported a relatively steady incidence of PDAC. On the contrary, the Dutch population-based study [10], covering only a twenty-year period from 1997 to 2016, found an ever-increasing incidence of PDAC from 12.1to 15.3 per 100,000 individuals, and the annual incidence of pancreatic cancer in Finland increased from 19 per 100,000 in 2000 to 24 per 100,000 in 2016 [21]. The present study showed an ever-increasing number of patients affected by PDAC from the period 2006–2010 (31.3%) to period C (39.3%) (*P* = 0.032). This finding indicated that better detection (in particular of, small lesions) and classification of tumours by means of improved diagnostic procedures was possible. Secondly, the major number of patients affected by pancreatic cancer in a tertiary referral centre could be the result of a centralisation policy. The body mass index, the comorbidity rate and KFS showed a decreasing incidence in periods B and C with respect to the period A. This indicated that, when the tumour was diagnosed in periods B and C, the patients were in better general health with respect to period A, probably because the symptoms related to the tumour were recognized early and an early diagnosis was possible. In addition, this finding could be related to the fact that a majority of the patients were fit for pancreatic resection. Finally, the proportion of patients receiving a biliary stent remained stable over the time. Indeed, our preferred approach to palliation of biliary obstruction is endoscopic stenting.

Regarding the imaging techniques, the present study showed that, the trends of all the diagnostic procedures, except for MR, changed over the time periods. First, a CT scan was the most frequent procedure performed over the time periods (more than 90% of the patients underwent a CT scan) and its incidence significantly increased from period A (89.4%) to period C (95.1%) (*P* < 0.001). These data indicated that a CT scan represented the best imaging technique for the diagnosis and staging of the disease, in particular owing to its reliability in assessing the involvement of the pancreatic vessels due to the pancreatic cancer. Nevertheless, of all the diagnostic procedures, EUS was the imaging technique which was found to be the most increased over the time periods (from 11.1% in period A to 49.4% in period C, *P* < 0.001), even if it remained a second level diagnostic tool as shown by its low incidence rate (range 10–50%). The increasing use of EUS was probably related to its usefulness in detecting small lesions better than a CT scan and in performing FNA/FNB (these procedures increased from 39.0% in period A to 57.7% in period C; *P* < 0.001). An FDG-PET/CT scan was performed in a few cases (approximately 10% of cases), confirming its value as a third level diagnostic procedure. However, its use significantly increased from 9.7% in period A to 16.5% in period C. This finding could be justified by the ever-increasing use of neoadjuvant therapy and by the need to compare the morphological and functional findings of the disease before and after the neoadjuvant therapy with the aim of properly defining the surgical indication. Finally, the use of CEUS decreased significantly from 15.6% in period A to 10.7% in period C (*P* = 0.028), probably because its usefulness was limited to only those selected cases in which there was a “CT scan doubt” regarding distant metastases to the liver.

Regarding staging, it should be noted that, in the total sample, 34.4% (stages IA, IB, IIA) were limited to the pancreas, 28.1% (Stages IIB, III) presented lymph node involvement and 37.5% (Stage IV) were metastatic. Over the time periods, a significant increase in early stages (stage 1A from 6.8% in period A to 11.6% in period C) was noted, probably related to better knowledge of the disease and improvement in diagnostic tools, capable of recognising small tumours.

Regarding the treatment of pancreatic cancer, throughout the entire study period, the majority of patients received only palliative treatment (61.0%), including mainly chemo- and radiotherapy, with an increasing incidence of palliative care over the time periods. This finding indicated that pancreatic cancer was most often diagnosed at an advanced stage, partially due to late presenting symptoms, and it has poor biological behaviour which could explain the negligible improvement in overall survival in the entire population. It is to underline that chemo and radiotherapy changed over the time. In particular, in the period 2006–2010, monochemotherapy was used (gemcitabine alone). Subsequently, polichemotherapy schedules as well as gemcitabine plus NAB-Paclitaxel, gemcitabine plus capecitabine and, finally, modified FOLFIRINOX (Leucovorin, 5-Fluorouracil, Irinotecan and Oxaliplatin) were adopted.

The use of up-front surgery, as a first therapeutic approach, decreased significantly over the time periods (from 39.2% in period A to 26.6% in period C; *P* < 0.001), possibly indicating (1) better preoperative evaluation and improved detection of non-resectable disease and (2) changes in trends of early therapeutic decision making with a decreasing rate of up-front surgery, probably in favour of neoadjuvant treatment which increased over the time periods from 6.4% in period A to 9.9% in period C. This finding confirmed the results reported by some nationwide studies [22, 23], suggesting that neoadjuvant treatment for pancreatic adenocarcinoma is only utilised in a minority of cases; however, its use appears to be increasing over time in both borderline and resectable tumours as has been recommended by various guidelines [2, 20]. The increasing use of neoadjuvant chemotherapy seems to be related with the major effectiveness of the new chemotherapeutic schedules, in particular those including more than one chemotherapic agent. R status changed over the time. In particular R0 status decreased from the first period (66.1%) to the last period (34.1%) and, at the same time, the R1 status increased from 33.1 to 64.4%. These data seem to be related with a more accurate study of the specimen by pathologists because surgical technique improved over the time. Mini-invasive approach increased over the time from 0 to 11.9%. This datum is clearly related to the learning curve of each surgeon. Exploratory laparotomy significantly decreased from 12.0% in period A to 4.7% in period C as, in recent years, the indications for surgery have been more carefully evaluated regarding both improvements in diagnostic procedures and the important support of the MDTB which includes radiologists, gastroenterologists, and oncologists with expertise in the pancreatic field. Finally, the MDTB evaluation of patients affected by PDAC increased significantly over the time periods from 32.6% in period A to 58.3% in period C. This finding indicated that the MDTB represented an essential instrument of effective cancer policy which allowed providing the highest quality care the patients needed and deserved. The MDTB could be considered to be the core component in organising cancer care and setting down the elements for guiding changes in trends over time. Postoperative outcomes were similar in the three periods. The increasing rate of CR-POPF could be related with a more accurate definition of a pancreatic fistula over the time. Even with all these changes in trends regarding pancreatic cancer, patient survival has not improved significantly in the past 15 years, even if the slight improvement in survival could be said to represent a trend (*P* = 0.097). However, a survival benefit of three months between period A (7 months)and period C (10 months) was not negligible as the majority of patients only received palliativecare, confirming not only the delayed diagnosis of the disease and its poor biological behaviour, but also the increasing effectiveness of the chemotherapeutic agents.

The present study had some limitations, namely the retrospective design from large databases which could potentially lead to coding error. In addition, the results of the study may have been affected by the different effectiveness of the imaging techniques over the time periods, by the improvement in pancreatic surgery, and the efficacy of chemo- and radiotherapy. The main strength of the study was the analysis of all patients affected by PDAC observed at S.Orsola-Malpighi Hospital, a tertiary referral centre.

In conclusion, despite its limitations, the present study showed the evolving knowledge in surgical oncology of pancreatic cancer at a tertiary referral centre over the time. The increasing incidence, the greater use of EUS and FNA/FNB, a decreasing rate of exploratory laparotomy and, finally, an important modification in management in favour of neoadjuvant treatments should be noted. All these changes were related to improvement in the diagnostics and therapeutic tools used to manage pancreatic cancer, and better knowledge of the disease which justified the different models of care for pancreatic cancer adopted over the time periods. Specifically, the introduction of the MDTB into the decision-making process and the institution of a pancreas unit seemed to ensure appropriate management of the disease showing that a system largely based on “a single man power” evolved in a real and useful teamwork.

## Data Availability

Data reported are available, included in our database of pancreatic cancer.

## Code availability

n.64/2017/U/Oss.
